# Phytochemicals as a Complement to Cancer Chemotherapy: Pharmacological Modulation of the Autophagy-Apoptosis Pathway

**DOI:** 10.3389/fphar.2021.639628

**Published:** 2021-05-07

**Authors:** Md. Ataur Rahman, Md. Abdul Hannan, Raju Dash, MD. Hasanur Rahman, Rokibul Islam, Md Jamal Uddin, Abdullah Al Mamun Sohag, Md. Habibur Rahman, Hyewhon Rhim

**Affiliations:** ^1^ Center for Neuroscience, Korea Institute of Science and Technology (KIST), Seoul, South Korea; ^2^ Global Biotechnology & Biomedical Research Network (GBBRN), Department of Biotechnology and Genetic Engineering, Faculty of Biological Sciences, Islamic University, Kushtia, Bangladesh; ^3^ Department of Anatomy, Dongguk University College of Medicine, Gyeongju, South Korea; ^4^ Department of Biochemistry and Molecular Biology, Bangladesh Agricultural University, Mymensingh, Bangladesh; ^5^ Department of Biotechnology and Genetic Engineering, Bangabandhu Sheikh Mujibur Rahman Science and Technology University, Gopalganj, Bangladesh; ^6^ ABEx Bio-Research Center, Dhaka, Bangladesh; ^7^ Department of Biotechnology and Genetic Engineering, Faculty of Biological Sciences, Islamic University, Kushtia, Bangladesh; ^8^ Department of Biochemistry, College of Medicine, Hallym University, Chuncheon-si, South Korea; ^9^ Graduate School of Pharmaceutical Sciences, College of Pharmacy, Ewha Womans University, Seoul, South Korea; ^10^ Department of Global Medical Science, Wonju College of Medicine, Yonsei University, Seoul, South Korea; ^11^ Division of Bio-Medical Science and Technology, KIST School, Korea University of Science and Technology (UST), Seoul, South Korea

**Keywords:** phytochemicals, pharmacology, apoptosis, autophagy, anticancer

## Abstract

Bioactive plant derived compounds are important for a wide range of therapeutic applications, and some display promising anticancer properties. Further evidence suggests that phytochemicals modulate autophagy and apoptosis, the two crucial cellular pathways involved in the underlying pathobiology of cancer development and regulation. Pharmacological targeting of autophagy and apoptosis signaling using phytochemicals therefore offers a promising strategy that is complementary to conventional cancer chemotherapy. In this review, we sought to highlight the molecular basis of the autophagic-apoptotic pathway to understand its implication in the pathobiology of cancer, and explore this fundamental cellular process as a druggable anticancer target. We also aimed to present recent advances and address the limitations faced in the therapeutic development of phytochemical-based anticancer drugs.

## Introduction

Cancer is responsible for 9.6 million deaths in 2018 and is listed as the second leading cause of death globally. Cancer thus poses a pivotal public health concern worldwide (WHO, 2018). During the 20th century, the cancer death rate was found to markedly increase, primarily because of abnormal lifestyles, such as excessive tobacco use (Siegel et al., 2020), physical and chemical carcinogens (Bhatia et al., 2020), alcohol use (Sanford et al., 2020), unhealthy diet (Khaltaev and Axelrod, 2020), and biological carcinogens (Hartwig et al., 2020). Delaying cancer treatment initiation increases patient mortality (Hanna et al., 2020). However, increased awareness about the need for lifestyle modification, early detection, and treatment may have contributed to a decline in cancer prevalence (i.e., by 1.5%, on average, per year from 2013 to 2017) (Henley et al., 2020). Cancer treatment options, such as chemotherapy, radiation therapy, hormone therapy, gene therapy, immunotherapy, photodynamic therapy, targeted therapy, surgery, palliative care, and a combination of these, are increasing in both number and efficiency across multiple types of cancer and for various patients (Markham et al., 2020). The main goal of cancer therapy is to stimulate the death of abnormal cells and preserve normal cells (Schirrmacher, 2019). Chemotherapy is the backbone of many cancer treatments. It aids in the reduction of tumor size and kills cancer cells at primary sites or metastasizing sites (Sak, 2012; Alfarouk et al., 2015). However, response to treatment varies substantially according to the type of cancer or even with the same type of cancer (Sak, 2012). Resistance to chemotherapeutic agents poses a major problem in cancer treatment, ultimately limiting the efficiency of anticancer drugs, which causes therapeutic failure and eventually death (Alfarouk et al., 2015). Chemotherapy resistance can be attributed to numerous mechanisms, including multi-drug resistance, alterations of cell death mechanisms (autophagy and apoptosis), changes in drug metabolism, epigenetic and drug targets, enhanced DNA repair and gene amplification, tumor cell heterogeneity, drug efflux and metabolism, and tumor microenvironment stress-induced genetic or epigenetic alterations as a cellular response to drug exposure (Wang et al., 2019). Among these mechanisms, alterations in autophagy (‘self-eating’) and apoptosis (‘self-killing’), which are two self-destructive processes that have propelled scientific innovation, are the vital causes of chemotherapy resistance (Thorburn et al., 2014). Autophagy, an evolutionarily conserved and regulated cellular recycling mechanism, has emerged as a key player in metabolic and therapeutic stresses. In fact, this mechanism attempts to maintain or restore metabolic homeostasis via the catabolic degradation of unnecessary proteins and injured or aged organelles (Santana-Codina et al., 2017). The role of autophagy in cancer treatment is paradoxical; it may act as a pro-survival or pro-death mechanism to counteract or mediate the cytotoxic effect of anticancer agents (Santana-Codina et al., 2017). Autophagy primarily functions as a tumor suppressor by modulating reactive oxygen species (ROS) within cells and maintaining genetic instability (Levine and Kroemer, 2008). Moreover, accumulating evidence suggests that faulty autophagy is linked to malignant transformation of cancer stem cells (Moosavi et al., 2018). Under these conditions, autophagy stimulation might be a critical approach to halt early tumor formation and development (Moosavi et al., 2018). However, autophagy can promote the growth and survival of current tumors during migration and epithelial-to-mesenchymal transition. Further, this process can help cancer stem cells escape immune surveillance and make cancer cells resistant to anoikis (Moosavi et al., 2018; Rahman et al., 2020). In this regard, inhibition of autophagy increases chemotherapy-induced cytotoxicity. Therefore, autophagy, a double-edge sword that works in a context-dependent manner, blocks the early stages of tumorigenesis while becoming a driver of tumor invasion and metastasis at later stages (Moosavi et al., 2018). The molecular mechanisms regulating the switch between these different modes of action are poorly understood (Kardideh et al., 2019). Nonetheless, the interplay between apoptosis and autophagy can be leveraged to improve cancer therapy (Tompkins and Thorburn, 2019). Cancer cells become chemotherapy-resistant by escaping some of the potential apoptotic mechanisms, such as downregulated pro-apoptotic signals, upregulated anti-apoptotic signals, and faulty apoptosis initiation and implementation. However, the functional relationship between apoptosis and autophagy is complex and has recently been deciphered at the molecular level. Therefore, modulating the key factors in the autophagic and apoptotic pathways may be a novel therapeutic strategy for enhancing chemotherapy efficiency.

The potential roles of phytochemicals in the modulation of autophagy and apoptosis have recently been reviewed (Deng et al., 2019). However, autophagy and apoptosis induction and/or inhibition are extremely complex processes that require thorough exploration. Nevertheless, a better understanding of the crosstalk between autophagy and apoptosis will enable further developments of novel anticancer therapeutic strategies. In this review, we summarize the molecular mechanisms of autophagy and apoptosis in cancer. Given the pivotal role of phytochemicals in cancer therapy, we sought to discuss various phytochemicals that could regulate autophagy and apoptosis-related signaling pathways to enhance cancer chemotherapy outcomes.

## Methods

A literature-based search was accomplished to collect published databases and relevant methodological contributions of the molecular mechanism of phytochemicals in autophagy-apoptosis modulation and cancer prevention has been conducted using PubMed, Scopus, Google Scholar, Web of Science, and Google that includes all original research articles written in English on multifunctional role of phytochemicals. Searching was conducted using various keywords including autophagy, apoptosis, natural compounds, cancer, phytochemical, neurodegenerative diseases, solid tumors and lymphomas, heart/cardiovascular diseases, perspectives role autophagy in cancer therapy and so on. All figures were generated using Adobe Illustrator software.

## Molecular Mechanism of Autophagy in Cancer

**FIGURE 1:**
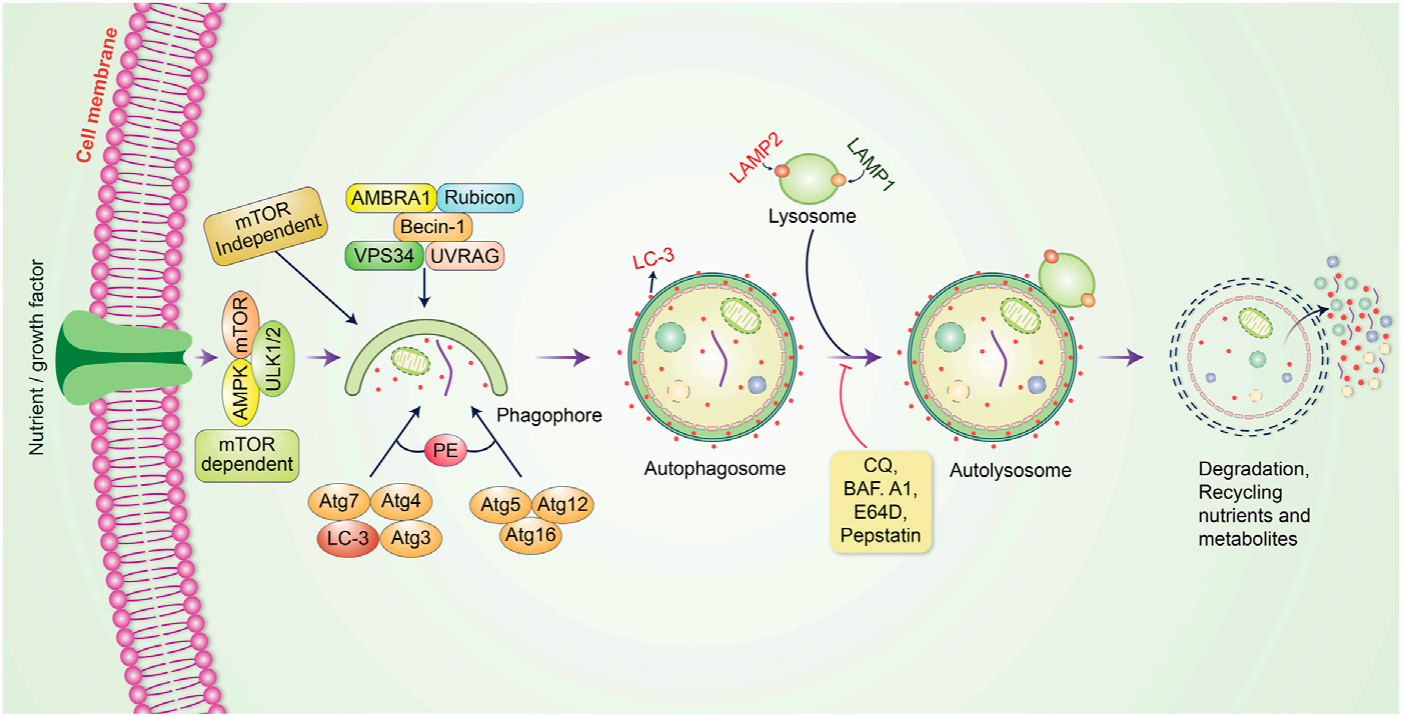
Molecular mechanism of the autophagic pathway. Autophagy is initiated by the formation of a pre-autophagosomal structure. PI3K-AMPK and mammalian target of rapamycin (mTOR) contribute to the formation of the pre-autophagosomal structure. ULK1, Vps34 and the Beclin-1 complex help to activate phagophore formation. After phagophore nucleation is elongated, subsequent binding to autophagosome occurs. Binding between mature autophagosome and lysosome results in autolysosome formation. Finally, autolysosomes are eliminated through acid hydrolases, which produce nutrients and recycling metabolites.

Autophagy is a cellular process that breaks down or degrades unwanted or aggregated dysfunctional cellular components through fusion with lysosomes; this cellular process is known to play an essential role in maintaining cellular function as well as homeostasis (Krishnan et al., 2020). Autophagy preserves an active interlink in cell defense as well as a cytostatic link in cancer cell progression (Rahman and Rhim, 2017). Generally, the process of autophagy might be introduced by the generation of pre-autophagosomal structures known as phagophore assembly sites (PAS) (Hurley and Young, 2017; Rahman and Rhim, 2017). Phosphatidylinositol 3-phosphate (PI3K), which is associated with the endoplasmic reticulum (ER), plays an essential role in the initiation of PAS formation (Kotani et al., 2018). AMP-activated protein kinase (AMPK), mammalian target of rapamycin (mTOR), and unc-51 like autophagy activating kinase-1 (ULK1) have been demonstrated to facilitate phagophore formation during autophagy induction (Alers et al., 2012), with Vps34, Vps15/p150, and Beclin-1 as recruiters for phagophore formation (Velazquez and Jackson, 2018). After phagophores are formed, phagocytosis occurs. This process is subsequently followed by expansion and sealing to elongate the membrane for autophagosome formation (Rubinsztein et al., 2012). Mature autophagosomes bind to lysosomes, resulting in autolysosome formation (Kardideh et al., 2019). Eventually, autolysosomes containing inner cargos are degraded by acid hydrolases and produce nutrients; other recycling metabolites subsequently preserve cellular homeostasis (Figure 1). The fate of cancer cells is thus dependent on autophagy (Wei and Huang, 2019). Autophagy decides whether the cancer is suppressed or promoted under certain conditions. mTOR plays an important role in protecting or activating oncogenic cells through the induction of autophagy. However, chemotherapy drugs have been found to suppress tumor cells by modulating autophagic pathways. Furthermore, inhibition of this pathway regulates cancer progression, and the influence of autophagy becomes either a cellular survival or death function (Jung et al., 2020). The metabolism of malignant cells is intensely altered to retain their proliferation and survival under adverse microenvironmental conditions. Autophagy plays an essential role in maintaining metabolic adaptations in cancer cells (Goldsmith et al., 2014). Although autophagy is recognized to sustain neoplastic cell metabolism under stress, the mutual association between cancer cell metabolism and autophagy remains unknown. mTOR and AMPK have been identified as the main signaling components that modulate autophagy via the regulation of amino acid and glucose levels (Alers et al., 2012). However, specific metabolites, ROS, growth factors, palmitate, oxygen concentration, ATP to ADP ratio, specific amino acid levels, and oncogenes regulate autophagy initiation and autophagosome formation. Further, they regulate this fine balance by assimilating these autophagy-related signals in cancer (Singh and Cuervo, 2011; Panda et al., 2015). Prominently, autophagy has been frequently identified to play a “dual role” as it can either hinder or stimulate cancer initiation and progression (Patra et al., 2020; Rahman et al., 2020a). In the present review, we outline the dual role of autophagy in tumorigenesis and emphasize our recent understanding of autophagy regulation of cancer cell activation and metabolism to control tumor growth and progression.

## Molecular Mechanism of Apoptosis in Cancer

**FIGURE 2:**
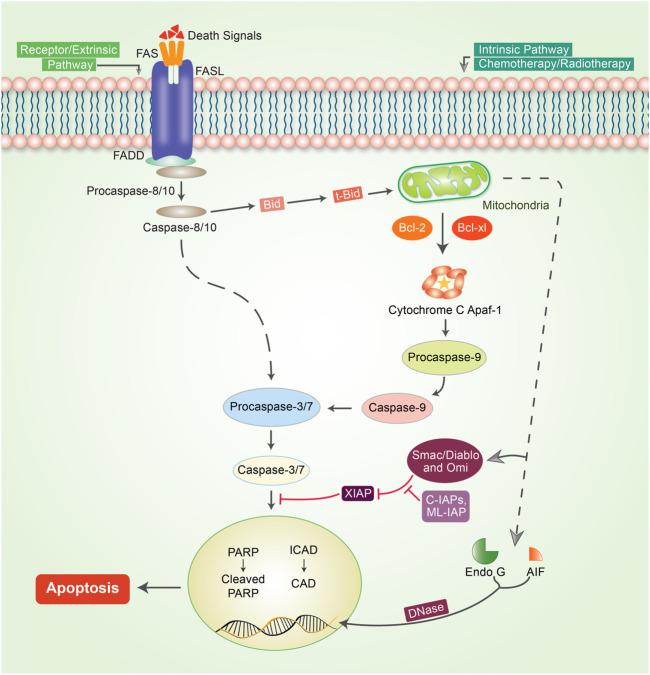
Mechanism of the apoptotic pathway in cancer. To initiate apoptosis, two central pathways are involved in this mechanism: the intrinsic pathway and extrinsic pathway. The extrinsic pathway of apoptosis is well defined by the TNF-α/TNFR1 and FasL/FasR models. Herein, the death receptor is induced by an adaptor protein; adaptor proteins are comprised of FADD (Fas-associated death domain) and TRADD (TNF receptor-associated death domain). The signaling that occurs through the extrinsic pathway causes the attachment of DRs to specific death ligands (DLs), thereby forming a death-inducing signaling cascade (DISC). The complex pathway of caspase-8 activation follows a predefined system that actively enables caspase-8 to detach from the DISC, whether or not the pro-domain of caspase-8 is retained as part of the DISC to initiate the signaling phases of apoptosis. However, in most apoptotic cells, proteins are customarily engaged in intrinsic phases that involve caspase-9, SMAC/DIABLO, Bcl-2, Bcl-w, Aven, Nox, and MYC. Mitochondrial dysfunction is followed by the loss of inner membrane mitochondrial potential, adequate formation of superoxide ions, impaired mitochondrial biogenesis formation, release of intra-membrane proteins, and matrix calcium glutathione burst, which enumerate the important potential for cancer therapeutic strategies by triggering the intrinsic phases of apoptosis in tumor cells. The execution phase of apoptosis initiator caspases, such as caspase-8/-9 or caspase-activated dnase (CAD), Poly (ADP-ribose polymerase (PARP), and other caspases such as caspase-3, -6, -7, and caspase-10, are typified as upregulator or executioner caspases. Caspase-3 is the most essential and effective of all effector caspases because it can be activated by all initiator caspases.

Apoptosis or programmed cell death is one of the predominant strategies for blocking or avoiding cancer or cancer formation (Lopez and Tait, 2015). Focusing on apoptosis is most effective for different cancer types because escaping apoptosis is a trademark of cancer and is indifferent to the type of cancer. Apoptosis is generally a central pathway that is associated with intrinsic and extrinsic pathways (Elmore, 2007). However, these extrinsic and intrinsic pathways could be involved in the same station, which is known as the execution pathway (Goldar et al., 2015) (Figure 2). To initiate apoptosis in apoptotic cells, the extrinsic pathway uses extracellular signals to induce apoptosis *via* stimulation of Fas ligand, tumor necrosis factor (TNF), and TNF-related apoptosis-inducing ligand (TRAIL), which interact with the extracellular transmembrane domain of death receptors (DR) (Guicciardi and Gores, 2009). Finally, caspases participate in the extrinsic pathway and are generally typified as starter, stimulator, or executioner caspases owing to their involvement and participation in the apoptotic signaling pathways. The intrinsic apoptotic pathway is directly involved in mitochondria-mediated proteins. Different stimuli, such as adequate Ca^2+^, impaired DNA molecules, oxidative stress (OS), surplus oxidants, deprivation of growth factors, and drug treatment and irradiation, have been associated with this pathway (Ghavami et al., 2004; Hassan et al., 2020). When Bax/Bak is incorporated into the mitochondrial membrane, it triggers the release of cytochrome c from the mitochondrial inner membrane into the cytosol (Kim, 2005). The intrinsic pathway of cell death is caused by Bcl-2 family proteins, which are pro-apoptotic and anti-apoptotic proteins, including Bcl-2 and Bcl-xL (Ghobrial et al., 2005). Apaf-1 and procaspase-9 combine with cytochrome c to form an apoptosome. Both mitochondria-dependent (intrinsic) and independent (extrinsic) pathways are connected at the same point, called the execution pathway (Elmore, 2007). The extrinsic and intrinsic phases are linked at the same point after caspase-8 is triggered. Activated caspase-8 in the extrinsic mechanism regulates the activation of BH3 interacting-domain (BID), a pro-apoptotic protein alternatively called BH3-only protein. BID then stimulates and oligomerizes the pro-apoptotic proteins, BAX and BAK, resulting in an intrinsic apoptotic phase (Green and Llambi, 2015).

## Phytochemicals Modulate Autophagy-Apoptosis Signaling in Several Cancers

**FIGURE 3:**
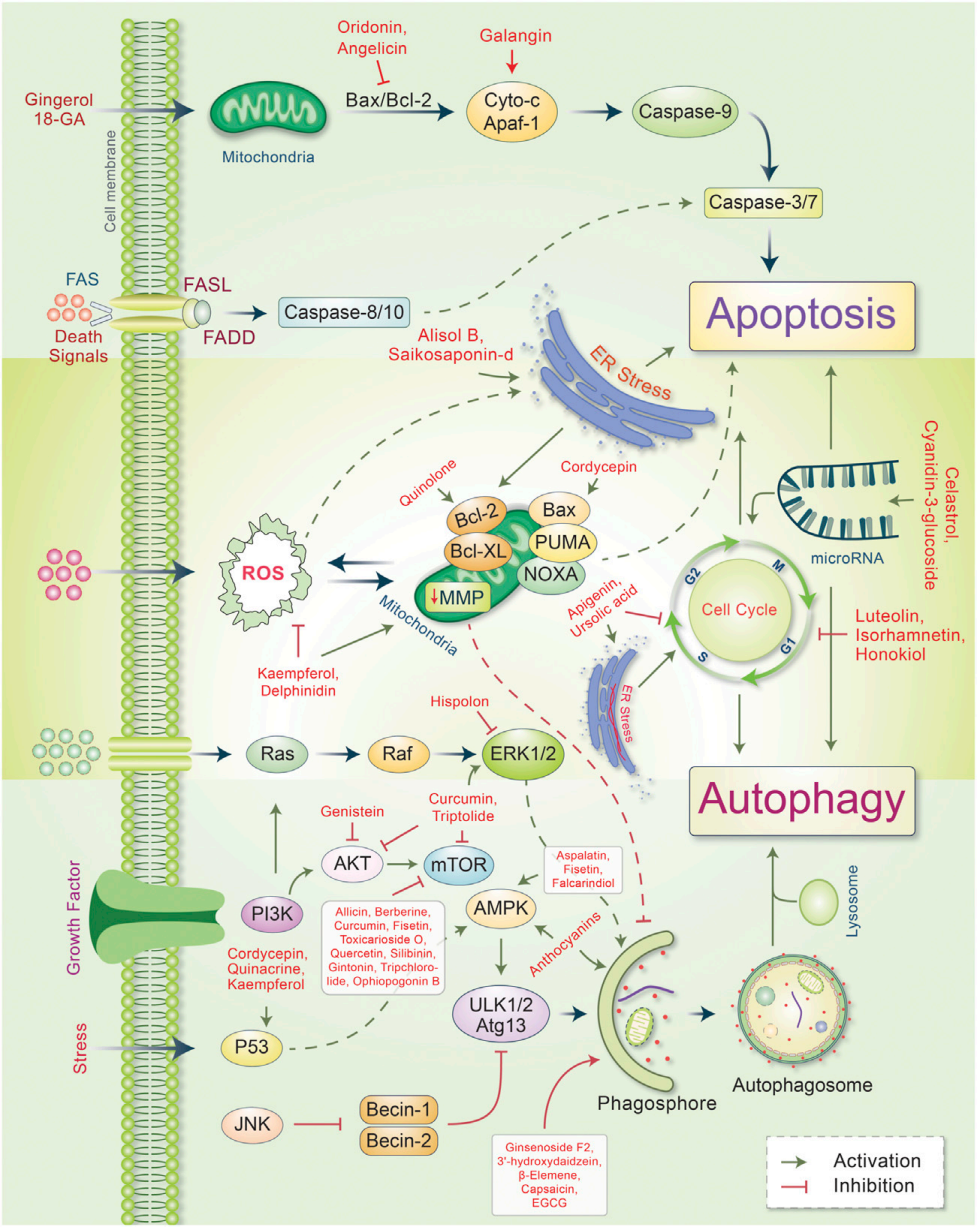
Major phytochemicals induce the signal transduction pathways that regulate autophagic and apoptotic cell death in cancer. Phytochemicals have been found to activate both the intrinsic and extrinsic apoptotic pathways by inducing a dysfunction in mitichrondria-caspase-9 and FAS-ligand-caspase-8 mediated apoptotic cell death, respectively. Phytochemicals induce ER stress and apoptotic cell death. However, some phytochemicals modulate mitichrondrial biogenesis and ensure apoptosis-autophagic cell death. Phytochemicals regulate the cell cycle and microRNA as well as cause apoptosis-autophagic cell death in cancer cells. Some phytochemicals activate autophagic signaling and inhibits cell growth and autophagy. For a detailed explanation, see the text.

Autophagy plays an essential role in cancer treatment, especially in chemotherapy, by removing dysfunctional organelles and intracellular components and inducing lysosomal degradation. This self-digestion mechanism strengthens cellular defense to protect cells from various intracellular and extracellular stresses and regulate redox balance to provide genomic and cytoplasmic stability. Emerging evidence supports the dual role of autophagy in cancer (i.e., as a promoter and an inhibitor of tumor development). However, the induction of autophagy in cancer is still a potential strategy; this is because it induces type II programmed cell death. During cancer initiation, autophagy regulators, such as mTOR and AMPK, are negatively modulated by tumor-suppressing factors, which cause autophagy induction (Comel et al., 2014). However, these autophagy regulators are activated by several oncogenes that suppress autophagy and promote cancer formation (Choi et al., 2013). Autophagy also suppresses carcinogenesis by regulating ROS, and excessive ROS production promotes tumor generation (Ávalos et al., 2014; Filomeni et al., 2015). Owing to their multifaceted therapeutic activities, phytochemicals have proven to be promising for treating many cancers (Mitra and Dash, 2018). In some cases, metabolites and synthetic products from natural compounds have demonstrated better chemopreventive effects than their original compounds (Aung et al., 2017). Our model and emerging evidence indicate that phytochemicals targeting the autophagic-apoptotic pathways are promising agents for cancer treatment for both pathways, or are dependent- and -independent of target-specific molecular mechanisms in cancer cells (Figure 3). Several phytochemicals and their autophagic-apoptotic effects are summarized in Table 1.

**TABLE 1:** Phytochemicals that activate autophagy and apoptosis in various *in vitro* and *in vivo* cancer models.

Phytochemicals	Doses/Conc	Cancer model	Molecular effects	References
Resveratrol	10–100 μM	Human colon carcinoma cell lines SW480, SW620, B103, and HCT116	Activate procaspase-3, 8/FADD	Delmas et al. (2003); Rahman et al. (2012a)
Eriocalyxin B (EriB)	1.4 μM	Human pancreatic cancer cellPANC-1, SW1990 CAPAN-2, and CAPAN-1	Caspase 8,9 activation and downstream regulation of caspases 3, 7, PARP	Li et al. (2012)
β-Elemene	10 μM	Human breast cancer cell lines Bcap37, MBA-MD-231	Conservation of LC3-I to LC3-II	Guan et al. (2014)
Oblongifolin C	15 μM	Human breast carcinoma cell lines HeLa or MEF	Activation of CASP3 and cleaved PARP	Lao et al. (2014)
Apigenin	10 μM	Colorectal cancer cells HCT-116, SW480, HT-29 and LoVo	Activate NAG-1, p53, p21	Zhong et al. (2010)
Allicin	1 μg/ml	Human gastric cancer cell line MGC-803, BGC-823 and SGC-7901	Increase expression of p38 and cleaved Of caspase 3	Zhang et al. (2015)
Anthocyanins	50 µM	Breast cancer cell lines MCF-MDA-MB-231 and MDA-MB-453	Inhibit the expression of VEGF, suppressed the MMP-9,MMP-2 and uPA expression	Hui et al. (2010)
Aspalathin	0.2 µM	Ovarian cancer cell Caov-3	Inhibit Dox, decrease expression of p53 and induce AMPK and Foxo1	Lin et al. (2017)
Baicalein	200 µM	Human HCC cell lines SMMC-772 and Bel-7402	Downregulate Bcl 2, increase ER stress	Wang et al. (2014)
Berberine	100 nM	Human glioma cell lines U251 and U87 GBM	Inhibition of AMPK/mTOR/ULK1	Peng et al. (2008); Wang et al. (2010); Yu et al. (2014); Guamán Ortiz et al. (2015); Wang et al. (2016a)
Capsaicin	150 µM	Human nasopharyngeal carcinoma cell line NPC-TW01	Downstream of PI3K/Akt/mTOR, increase caspase-3 activity	Lin et al. (2017b)
Celastrol	1.5 μM	Human prostate cancer cell lines LNCaP, 22Rv1, DU145 and PC-3	Upstream of miR-101	Guo et al. (2015)
Cordycepin	200 µM	Human brain cancer cellSH-SY5Y and U-251	Upregulates ROS, p53, and LC3II	Chaicharoenaudomrung et al. (2018)
Curcumin	25 µM	Malignant mesotheloma cancer cell line MM-B1, H-Meso-1, and MM-F1	Increase Bax/bcl-2 ratio, p53 expression, activation of caspase 9, cleavage of PARP-1	Masuelli et al. (2017)
Epigallocatechin gallate (EGCG)	100 nM	Vascular endothelial cell line U-937	Reduce TNF-α, inhibit VCAM1, LC3A, LC3B	Yamagata et al. (2015)
Evodiamine	10 µM	Gastric cancer cell line SGC-7901	Activates beclin-2, Bax, downregulates Bcl-2	Rasul et al. (2012)
Fisetin	40–120 µM	Prostate cancer cell lines PC3 and DU145	Supressed Mtor and inhibit Akt, activate AMPK	Suh et al. (2010)
Genistein	50–100 µM	Ovarian cancer cell line A2780	Reduces Akt/mTOR phosphorylation	Gossner et al. (2007)
Gingerol	300 µM	Human colon cancer cell lines SW-480 and HCT116	Inhibition of JNK, ERK1-2, and P38 MAPK	Shukla and Singh, (2007); Baliga et al. (2011); Radhakrishnan et al. (2014)
Ginsenoside F2	100 µM	Breast cancer cell lines MCF-7	Elevated Atg-7	Mai et al. (2012)
			Cleaved PARP	
Hispolon	25–100 µM	Cervical cancer cell lines Hela and SiHa	Downregulated lysosomal protease Cathepsin S(CTSS)	Chen et al. (2012)
3′-hydroxydaidzein (3′-ODI)	100 µM	Mouse melanoma cell line B16F1	Reduce the α-MSH	Kim et al. (2013)
Toxicarioside O	50 nM	Human colorectal cancer cell lines HCT116 and SW480	Inhibition of the Akt/mTOR	Huang et al. (2017)
			Upstream SIRT1↑	
Falcarindiol	6 µM	Human breast cancer cell lines MDA-MB-231,MDA-MB-468 and Her2	FAD induce expression of GRP78	Minto and Blacklock, (2008); Jin et al. (2012); Lu et al. (2017)
Oleanolic acid	100 μg/ml	Human pancreatic cancer cell line Panc‐28	Modulate JNK and mTOR pathway	Pollier and Goossens, (2012); Liu et al. (2014)
Honokiol	40 μM	Human glioblastoma cell lines LN229, GBM8401 and U373	Reduction of p-PI3K, p-Akt and Ki67	Cheng et al. (2016)
Magnolol	40 μM	Human glioblastoma cell lines LN229, GBM8401 and U373	Reduction of p-PI3K, p-Akt and Ki67	Cheng et al. (2016)
Alisol B	30 μM	Breast cancer cell lines MCF-7, SK-BR-3, and HeLa	Activation of Ca2+/AMPK/Mtor	Law et al. (2010)
Luteolin	100 µM	Human liver cancer SMMC-7721	Increase expression of caspase-8, decrease bcl-2	Cao et al. (2017)
α-Mangostin	5–10 µM	Human brain cancer cell lines, GBM8401 and DBTRG05MG	Activation of AMPK	Chao et al. (2011)
Oridonin	8–32 μmol/L	Human hepatocellular carcinoma cell line BEL-7402	Activation of caspase-3	Zhang et al. (2006)
			Down-regulation of Bcl-2 and Up-regulation of Bax	
Quercetin	15 µM	Lymphoma cell lines BC3, BCBL1 and BC1	Inhibits PI3K/Akt/mTOR and Wnt/β-catenin	Granato et al. (2017)
Rottlerin	1–2 µM	Breast cancer cell lines CD44/CD24	Enhance expression of LC3	Kumar et al. (2013)
6-Shogaol	55.4 μM	Lung cancer cell line A549	Inhibition af Akt and mTOR downstream	Hung et al. (2009)
Silibinin (silybin)	50 µM	RCC cell lines ACHN and 786-O	Inhibit mTOR and activate AMPK	Li et al. (2015)
Sulforaphane	40 µM	Human pancreatic cancer cell lines MIA PaCa-2,Panc-1	Increase ROS level	Naumann et al. (2011)
γ-tocotrienol	10 μmol/L	Breast cancer cell lines MCF-7 and MDA-MB-231	Activate AMPK, down regulate Ang-1/Tie-2	Ling et al. (2012); Tang et al. (2019)
Thymoquinone	40–60 µM	Oral cancer cell lines SASVO3,SCC-4, OCT,SAS	Increase expression of LC3-II, Bax expression	Chu et al. (2014)
Tripchlorolide	200 nM	Lung cancer cell line A549/DDP	Inhibition of PI3K/Akt/mTOR	Chen et al. (2017a)
Tetrandrine	0–4 μM	Hepatocellular carcinoma cell lines Huh7, HCCLM9 and Hep3B	Inhibits Wnt/β-catenin	Zhang et al. (2018)
			Decreases MTA1	
N-desmethyldauricine	150 μM	Lung cancer cell line H1299	Inhibition of Ulk-1/PERK/AMPK/mTOR	Law et al. (2017)
Quinacrine	15 μM	Colon cancer cell lines HCT-116/HCT-116/HCT-116	Activation of p53, p21, and inhibition of topoisomerase	Mohapatra et al. (2012)
Chloroquine	50 μM	Pancreatic cancer cell line MiaPaCa2 and S2VP10	Decrease the level of O_2_	Frieboes et al. (2014)
Tangeritin	10 μM	Breast cancer cell lines MCF7, MDA–MB–468 and MCF10A	Induce CYP1 and CYP1A1/CYP1B1 protein expression	Surichan et al. (2018)
Myricetin	100 μM/L	Prostate cancer cell lines PC3, DU145	Knockdown the interaction between P1M1/CXCR4	Ye et al. (2018)
Galangin	15 μM	Human kidney cancer cell line A498	Inhibition of PI3K/Akt/mTOR signaling	Zhu et al. (2018)
Isorhamnetin	100 μM	Colon cancer cell lines HCT116 and SW480	Increase ROS	Wu et al. (2018)
Hesperetin	350 μM	Lung cancer cell line H522	Knockdown caspase-3/9,p53,Bax	Elango et al. (2018)
			Upregulate Fas, FADD and caspase-8	
Delphinidin	80 μM	Breast cancer cell lines MDA-MB-453 and BT474	Suppression of mTOR	Chen et al. (2018)
			Activation of the AMPK	
Epigallocatechingallate (EGCG)	500 μM	Human glioblastoma cell lines T98G and U87MG	Increase ROS	Grube et al. (2018)
Epicatechin-3-*O*-gallate (ECG)	36 µM	Prostate cancer cell lines LNCaP and PC-3	Diminished the progression of carcinofenic cell	Siddiqui et al. (2011); Stadlbauer et al. (2018)
Cyanidin‐3‐glucoside (C3G)	20 μM	Human breast cancer MDA‐MB‐231 and Hs‐578T	Inhibiting STAT3/VEGF and *miR*124 mediated downregulation STAT3	Ma and Ning, (2019)
Benzyl isothiocyanate (BITC)	6.5 μM	Pancreatic cell lines BxPC-3 and PanC-1	Decrease the phosphorylation of PI3K/Akt/FOXO1/PDK1/mTOR/FOXO3a	Boreddy et al. (2011)
Phenethyl isothiocyanates (PEITC)	10 μM	Breast cancer cell lines MDA-MB-231 and MCF-7	Reduction of HER2, EGFR and STAT3 expression	Gupta and Srivastava, (2012)
Piperlongumine (PL)	6 µM	Lung cancer cell lines A549 and A549/DTX	Regulate PI3K/Akt/mTOR	Bezerra et al. (2008); Raj et al. (2011); Wang et al. (2015)
Saikosaponin-d	10 µM	Breast cancer cell lines HeLa and MCF-7	Calcium mobilization, induce CaMKKβ-AMPK-mTOR	Hsu et al. (2004); Tundis et al. (2009); Wong et al. (2013)
Guttiferone K	20 µM	Human HCCs HuH7 and HepG2	Reduce phosphorylation of Akt/mTOR, increase ROS	Xu et al. (2008)
				Wu et al. (2015)
Licochalcone A	20 or 50 µM	Breast cancer cell line MCF-7	Suppression of PI3K/Akt/mTOR pathway	Xue et al. (2018)
Ophiopogonin B	10 μM	Lung cancer (NSCLC) cell lines NCI-H157 and NCI-H460	Inhibition of PI3K, Akt, mTOR	Chen et al. (2013a)
Norcantharidin	40 μM	Human MHCC-97H (97H) and HepG2 HCC cells	Inhibition of c-Met, mTOR	Sun et al. (2017a)
Juglanin	10 μM	Breast cancer cell lines MCF-7 and SKBR3	Regulation of ROS, JNK	Sun et al. (2017b)
Isoliquiritigenin	25 μM	Human ovarian cancer cell lines, OVCAR5 and ES-2	Cleaved caspase-3, increased LC3B-II, and Beclin-1 level	Chen et al. (2017b)
Cucurbitacin B	200 μM	Breast cancer cell line MCF-7	Increase γH_2_AX, phosphorylation of ATM/ATR, ROS	Chen et al. (2005); Ren et al. (2015)
Carnosol	25 µM	Human breast cancer cell line MDA-MB-231	Increase p21/WAF1 and downregulate p27	Al Dhaheri et al. (2014)
Kaempferol	50 or 100 μM	Colorectal cancer cell lines HCT116, HCT15, and SW480	Generated ROS and p53 signal	Choi et al. (2018)
Ursolic acid	10–40 µM	Prostate cancer cell lines PC3	Increases Beclin-1/Atg5 and inhibits Akt/mTOR	Shin et al. (2012)
Triptolide	200 nM	Human pancreatic cancer cell line S2-013, S2-VP10, and Hs766T	Inhibits of Akt-mTOR-P70S6K	Mujumdar et al. (2010)

### Phytochemicals in Autophagy Signaling

Apigenin is a flavonoid derivative that modulates several kinase pathways and inhibits the cell cycle at the G2/M phase. Studies have shown that apigenin can inhibit cell growth and induce autophagy in time-and dose-dependent manners in HepG2 cells (Zhong et al., 2010). Autophagy was also found to be mediated *via* the inhibition of the PI3K/Akt/mTOR pathway in HepG2 cells (Yang et al., 2018). An organic sulfur compound, allicin, acts as an antitumor agent that activates autophagic cell death by inhibiting the PI3K/mTOR signaling pathway (Sak, 2012). Allicin also inhibits the expression of p53 and Bcl-2, and upregulates the Beclin-1 signaling and AMPK/TSC2 signaling pathways (Chu et al., 2012). Anthocyanins (ACNs) present in black soybeans induce autophagy; however, their underlying mechanism have yet to be determined (Choe et al., 2012). Aspalathin is a polyphenolic dihydrochalcone C-glucoside that plays a critical role in inhibiting Dox-induced cardiotoxicity and decreasing P53 expression. Aspalatin triggered autophagy-related genes and decreased p62 by inducing the AMPK and Fox pathways (Johnson et al., 2017). Berberine is an isoquinoline alkaloid that exerts anticancer activity for autophagy induction by inhibiting the AMPK/mTOR/ULK1 pathway (Wang et al., 2016a). Celastrol is another triterpenoid that is effective against human prostate cancer. Celastrol blocks the AR signaling pathway, which induces autophagy and downregulates the expression of miR-101 (Guo et al., 2015). Cordycepin generates ROS in cancer cells and enhances p53 and LC3I/II expression, thereby modulating autophagy (Chaicharoenaudomrung et al., 2018). Cordycepin inhibits renal carcinoma in the migration of the Caki-1 cell line by reducing microRNA-21 expression and Akt phosphorylation, and increasing PTEN phosphatase levels (Yang et al., 2017). In addition, cordycepin induces autophagy *via* Bax activation in ovarian cancer cell lines, including SKOV-3 and OVCAR-3 (Jang et al., 2019). Curcumin has been shown to increase ROS and DNA damage in cancer cells. Further, curcumin increased the phosphorylation of ERK1/2 and p38 MAPK, inhibited Akt and P54 JNK (Masuelli et al., 2017), and eventually induced autophagy in NSLCA549 cells (Liu et al., 2018). Evodiamine, a quinolone alkaloid, mediates autophagy activation by upregulating Beclin-1 and Bax expression and downregulating Bcl-2 (Rasul et al., 2012). Fisetin is a naturally occurring flavonoid that is reported to suppress the mTOR signaling pathway *via* the inhibition of Akt and activation of AMPK, and autophagic programmed cell death in prostate cancer cells (Suh et al., 2010). Similarly, genistein displayed chemopreventive and chemotherapeutic effects in cancer cells. Treating ovarian cancer cells with genistein led to a reduction in Akt phosphorylation and induced autophagy, thereby contributing to glucose uptake reduction in cancer cells (Gossner et al., 2007). Ginsenoside F2 showed anti-proliferative activity and initiated the autophagic process in breast cancer stem cells. Concurrently, ginsenoside F2 elevated Atg-7 levels, induced the formation of acidic vascular organelles, and recruited GFP-tagged LC3-II to autophagosomes (Mai et al., 2012). Hispolon, a phenolic compound isolated from *Phellinus igniarius* (L.) Quél., exhibited apoptotic and anti-tumor effects in cervical cancer cell lines and notably induced autophagy. Treatment with hispolon inhibited metastasis by downregulating lysosomal protease cathepsin S (CTSS) (Chen et al., 2012). Further, hispolon was found to mechanistically block the ERK pathway and enhance LC3 conversion and acidic vesicular organelle formation (Hsin et al., 2017). 3′-hydroxydaidzein (3′-ODI) is another phytochemical derivative that induces autophagy. In fact, it was found to significantly reduce α-MSH-mediated melanogenesis in melanoma cells (Kim et al., 2013). Toxicarioside O, a natural product derived from the *Antira toxicaria* Lesch., showed anticancer potency through autophagy induction *via* the subsequent reduction of the Akt/mTOR pathway (Huang et al., 2017). Falcarindiol (FAD), a natural polyene (Minto and Blacklock, 2008) promotes autophagy in response to ER stress (Jin et al., 2012) while α-mangostin mediates autophagic cell death *via* AMPK activation in human glioblastoma cells (Chao et al., 2011). The bioflavonoid, quercetin, possesses anticancer and anti-inflammatory properties. In hyperactive primary effusion lymphoma (PEL), quercetin reduced the release of cytokines and inhibited PI3K/Akt/mTOR and STAT3 pathway-induced autophagy, ultimately resulting in PEL cell death (Granato et al., 2017). In breast cancer steam cells, rottlerin (Rott) enhanced the expression of LC3, Beclin-1, and Atg12 aggregation during autophagy. Silibinin (silybin) is a chemoprotective flavonoid that might exhibit anti-metastatic effects on renal cell carcinoma (RCC). Silibinin increased the expression of LC3-II, which not only suppressed mTOR regulation but also activated the AMPK pathway (Li et al., 2015). Sulforaphane (SFN) is a group of phytochemicals that are referred to as isothiocyanates (Uddin et al., 2020). Multiple studies have shown that autophagy in SFN-induced cell death eliminates highly resistant pancreatic carcinoma cells by releasing ROS, without exhibiting cytotoxic effects (Naumann et al., 2011; Uddin et al., 2020). Gintonin has been found to stimulate autophagic flux *via* the Akt/mTOR/p70S6K-mediated pathway in primary cortical astrocytes (Rahman et al., 2020b). Ursolic acid (UA), a pentacyclic triterpenoid, showed anti-proliferative effects *via* G1 phase arrest and induced autophagy regulation through the beclin-1 and Akt/mTOR pathways (Shin et al., 2012). Tripchlorolide is present in tripterygium. Treatment with tripchlorolide was found to attenuate the expression of the PI3K/Akt/mTOR signaling pathway (Chen et al., 2017a). Tetrandrine is a bisbenzylisoquinoline alkaloid isolated from the Chinese medicinal herb, *Stephania tetrandra* S. Moore. Tetrandrine plays an important role in the suppression of human hepatocellular carcinoma, inhibits the Wnt/β-catenin pathway, and reduces MTA1 expression, which eventually causes autophagy (Zhang et al., 2018). N-desmethyldauricine is a novel inducer of autophagy that is mediated by the inhibition of Ulk-1/PERK/AMPK mTOR and causes calcium accumulation, leading to autophagic cell death (Law et al., 2017). Quinacrine displayed anticancer properties in breast cancer cells by enhancing p53 and p21 regulation and inhibiting topoisomerase activity (Mohapatra et al., 2012). The anti-proliferative activity of tangeritin initiates anticancer activity by modulating autophagy and inducing the CYP1 enzyme and CYP1A1/CYP1B1 proteins in MDA-MB-468 and MCF-7 cells (Surichan et al., 2018). Multiple studies have indicated that licochalcone A treatment activates the LC3-II signaling pathway and suppresses the PI3K/Akt/mTOR pathway to promote autophagy in MCF-7 cells (Xue et al., 2018). In addition, ophiopogonin B was found to induce autophagy by inhibiting the PI3K/Akt/mTOR signaling pathway (Chen et al., 2013a). Anticancer activity was also exhibited by juglanin, which is generally extracted from green husks. Juglanin-mediated treatment attenuated G2/M phase arrest and induced autophagy by regulating the ROS/JNK signaling pathway in human breast cancer (Sun et al., 2017a). Cucurbitacin B (Cuc B) is another natural tetracyclic triterpene compound that is generally used as an anti-inflammatory drug (Chen et al., 2005). Treatment with Cuc B increases γH_2_AX protein expression, promotes DNA damage through phosphorylation of ATM/ATR, and concurrently increases the level of ROS that induces autophagy in MCF-7 cells (Ren et al., 2015).

### Phytochemicals in Apoptosis Signaling

*Angelica polymorpha* Maxim, which contains angelicin, increases cellular cytotoxicity and induces apoptosis by decreasing the expression of anti-apoptotic proteins, including Bcl-xL, Bcl-2, and Mcl-1 in SH-SY5Y human neuroblastoma cells (Rahman et al., 2012b; Rahman et al., 2012b). As FAD-induced cell death is known to be caused by caspase-dependent modulation, FAD is suggested to have a synergistic effect on several approved cancer drugs designed to kill cancer cells (Lu et al., 2017). Alisol B induces autophagy by modulating the CaMKK-AMPK-mTOR signaling pathway, calcium mobilization, and enhanced ER stress, leading to apoptotic cell death (Law et al., 2010). Luteolin is a flavonoid found in various plants and is known to play a leading role in hepatocellular carcinoma cell lines through G0/G1 phase cell cycle arrest. Studies have shown that treatment with luteolin induces apoptosis by increasing caspase-8 expression, reducing Bcl-2 at the mRNA level, improving the conversion of LC3B-I to LC3B-II, and decreasing the viability of SMMC-7721 cells (Cao et al., 2017). In the human carcinoma BEL-7402 cell line, oridonin-mediated apoptosis was found to be driven by the activation of caspase-3 as well as reduced Bcl-2 expression and Bax upregulation, which can inhibit cell growth (Zhang et al., 2006). Prolonged treatment with Rott in breast CSCs suppressed the phosphorylation of Akt and mTOR, and upregulated the phosphorylation of AMPK, eventually upregulating apoptosis (Kumar et al., 2013). Several natural plant extracts derived from *Dioscorea nipponica* Makino, *Melandrium firmum* (Sieb. & Zucc.) Rohrb., and *Saussurea lappa* (Decne.) Sch. Bip. have been found to induce anti-proliferative effects and apoptotic cell death in human neuroblastoma cells (Rahman et al., 2013; Rahman et al., 2014; Rahman et al., 2015). γ-Tocotrienol, a vitamin E isomer (Ling et al., 2012), is known to target Ang-1/Tie-2 and exert anti-cancer effects through the activation of AMPK signaling, leading to apoptotic cell death in human prostate cancer cell lines (Tang et al., 2019). Triptolide induced apoptosis in pancreatic cancer cells, causing the inactivation of Akt/mTOR/p70S6K and upregulation of the ERK1/2 pathway (Mujumdar et al., 2010). Kaempferol is a flavonoid compound that generates ROS and p53 signals and regulates p38 phosphorylation as well as caspase activation, thereby inducing apoptosis of colorectal cancer cells (Choi et al., 2018). Myricetin is a natural flavonoid found in various fruits and vegetables. A previous report suggested that myricetin attenuated tumor cell growth by promoting apoptotic cell death (Cao et al., 2018). Myricetin exerts pro-apoptotic and cytotoxic effects on prostate cancer cells by inhibiting P1M1 and downregulating the interaction between P1M1 and CXCR4 (Ye et al., 2018). Galangin induced apoptosis in kidney cancer cells by increasing the expression of Bax and Cyt-c and decreasing Bcl-2 expression (Zhu et al., 2018). In a human breast cancer cell line, isorhamnetin inhibited tumor growth by inducing cell cycle arrest in the S-phase and displayed strong cytotoxic effects *via* the ROS-dependent apoptotic pathway (Wu et al., 2018). In H522 cells, Hesperet induced apoptotic cell death by downregulating caspase-3/9, p53, and Bax expression and upregulating Fas, FADD, and caspase-8 expression (Elango et al., 2018). Cyanidin-3-glucoside (C3G) is an ACN found in fruits. C3G exerts anti-inflammatory properties and induces *miR*-124 expression. Concurrently, *miR*-124 regulation downregulates STAT3 and inhibits angiogenesis induced by C3G in human breast cancer (Ma and Ning, 2019). Benzyl isothiocyanate (BITC) is present in cruciferous vegetables. Administering BITC to mice caused decreased phosphorylation of PI3K/Akt/FOXO1/PDK1/mTOR/FOXO3a, which suppressed pancreatic cancer cell growth and induced apoptosis (Boreddy et al., 2011). Several studies have reported that glucosinolate-derived phenethyl isothiocyanates (PEITC) are promising anti-tumorigenic agents. In fact, PEITC-treated mice were found to exhibit reduced expression of HER2, EGFR, and STAT3, and enhanced apoptosis through the cleavage of caspase 3 and PARP (Gupta and Srivastava, 2012). NCTD inhibits c-Met and mTOR and exhibits anticancer properties (Sun et al., 2017b).

### Phytochemicals in Autophagic-Apoptotic Signaling

**FIGURE 4:**
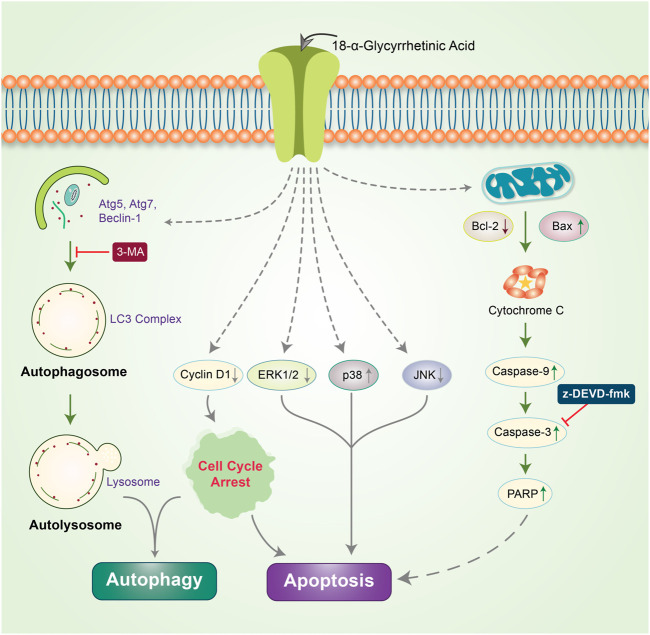
Anticancer effects of 18-GA in autophagy-apoptosis modulation in neuroblastoma cells. 18-GA encouraged caspase-induced apoptosis by depolarizing the mitochondria membrane potential (MMP). 18-GA also induced early autophagy through Atg5 and Atg7 activation and converted LC3I to LC3II. The autophagy inhibitor, 3-MA, inhibited 18-GA-mediated autophagy. Nonetheless, 18-GA caused the downregulation of ERK1/2, JNK, and cyclinD1 protein and the upregulation of p38 MAPK, which activated apoptosis in neuroblastoma cancer.

β-Elemene is a natural chemical compound collected from different medicinal plants, such as Curcuma WenYuJin (Edris, 2009). β-Elemene exerts cytoprotective activity by converting LC3-I into LC3-II to form autolysosomes that activate autophagy and significantly reduce the *in vitro* growth of human breast cancer cells *via* apoptosis (Guan et al., 2014). Capsaicin is another naturally occurring phytochemical that exerts antitumor potency by downregulating the PI3K/Akt/mTOR pathway. Capsaicin instigates the autophagy process by increasing the expression of the autophagy markers, LC3-II and Atg5, and enhances the degradation of p62 and Fap-1, while increasing caspase-3 activity (Lin et al., 2017a). The *Morus alba* L. root extract containing oxyresveratrol was previously found to accumulate ROS and induce autophagic and apoptotic cell death *via* the FOXO-Caspase-3 pathway in human neuroblastoma cells (Kwon et al., 2015; Rahman et al., 2017). Gingerol possesses antioxidant, anti-inflammatory, and anti-tumor properties (Shukla and Singh, 2007; Baliga et al., 2011), and inhibits colon cancer cell proliferation by activating the caspase-dependent pathway and concurrently cleaving PARP, which induces autophagy (Radhakrishnan et al., 2014). Concurrent treatment with honokiol (Hono) and magnolol (Mag) decreased the expression of cyclin A, D1, and cyclin-dependent kinase, which arrests cell cycle progression and reduces p-PI3K, p-Akt, and Ki67 expression in U87MG and LN229 human glioma cells. Both Hono-and Mag-mediated treatments exert synergistic anti-tumor effects by inhibiting cell proliferation. Accordingly, they induce autophagy and apoptosis in human GMB cells (Cheng et al., 2016). 6-Shogaol disrupts the Akt/mTOR mediated signaling pathway; blocking of Akt is beneficial to apoptotic cell death. 6-Shogaol induces autophagy through the inhibition of Akt overexpression and exhibits anticancer activity against non-small cell lung cancer (Hung et al., 2009). Thymoquinone (TQ), a major component of black cumin, exhibits potent cytotoxic effects in several cancer cell lines. In SASVO3 cells, TQ was found to mediate cell death caused by the enhancement of Bax expression and increase autophagic vacuoles and LC3-II protein expression following apoptosis and autophagy (Chu et al., 2014). In our previously published study, we revealed that the gap-junction inhibitor, 18α-Glycyrrhetinic acid (18-GA), induces apoptosis and autophagy. 18-GA-induced autophagy has been shown to induce Atg5, Atg7, and LC3II accumulation through p62 degradation (Rahman et al., 2016b). 18-GA was also found to destabilize the Bcl-2/Beclin-1 interaction and the cleavage of Beclin-1, ultimately highlighting the occurrence of mitochondrial-mediated apoptosis in SH-SY5Y cells (Figure 4). 18-GA is also known to activate several MAPKs and arrest the cell cycle, which leads to the activation of apoptosis. 18-GA may thus be used as a therapeutic target for the apoptosis-autophagy pathway in neuroblastoma.

**FIGURE 5:**
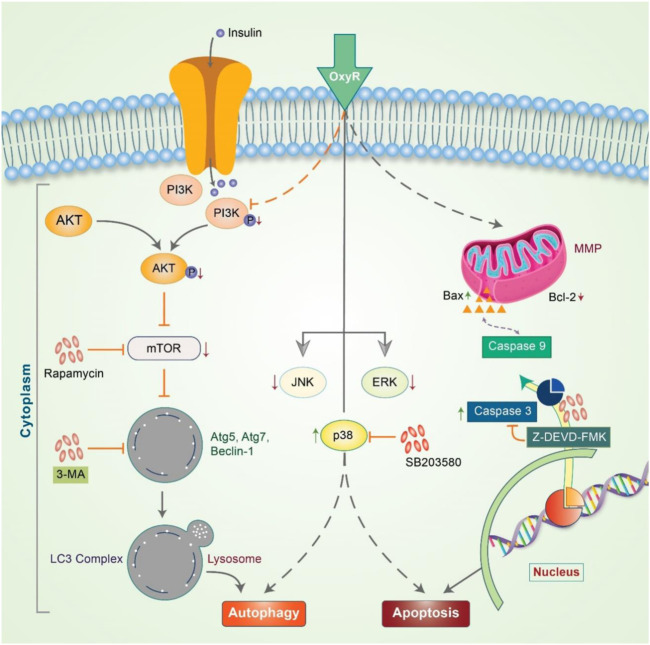
Oxyresveratrol controls the autophagy-apoptosis signal to modulate neuroblastoma cells. OxyR activates PI3K/Akt/mTOR and the inhibition of mTOR by rapamycin blocks autophagy, indicating an mTOR-dependent autophagic pathway. OxyR led to arrest at the G2/M phase of the cell cycle and activated mitochondria-mediated caspase-3 dependent apoptosis. OxyR was also revealed to increase Bax/Bcl-2 ratio without generating ROS or activating p53. When the p38 inhibitor, SB203580, was applied, OxyR was found to activate autophagy-apoptosis signaling in neuroblastoma cells.

Delphinidin is an anthocyanidin monomer with strong anti-oxidative characteristics. In HER-2 positive breast cancer cells, delphinidin enhances apoptosis and autophagy by suppressing mTOR and activating the AMPK signaling pathway (Chen et al., 2018). Emerging evidence has shown that epicatechin-3-O-gallate (EGCG) promotes autophagy and apoptosis in different cancer lines (Siddiqui et al., 2011; Grube et al., 2018; Stadlbauer et al., 2018). Previously, OxyR was found to simultaneously activate apoptosis and autophagy in NB. OxyR also reduces PI3K/Akt/mTOR signaling and enhances cytotoxicity by increasing autophagy levels (Figure 5) (Rahman et al., 2017). OxyR-induced cell death was found to occur independent of apoptosis induction due to alterations in the levels of PI3K/Akt/mTOR and p38 MAPK activity in SH-SY5Y cells.

Saikosaponin-d is reported to induce intracellular calcium accumulation and autophagy by activating the CaMKKβ-AMPK-mTOR pathway. Nonetheless, ER stress and UPR activation by saikosaponin-d have been demonstrated to trigger apoptosis and autophagic cell death (Wong et al., 2013). Isoliquiritigenin (ISL) hinders the viability of ovarian cancer cell lines (OVCAR5) and the ES-2 model. ISL also induced autophagy in OVCAR5 *via* cell cycle arrest at the G2/M phase, cleaved caspase-3, and increased LC3B-II and Beclin-1 expression (Chen et al., 2017b). Guttiferone K (GUTK) isolated from *garcinia yunnanensis* Hu (Xu et al., 2008) was found to reduce Akt phosphorylation and inhibit the mTOR pathway. GUTK also enhanced ROS and triggered the phosphorylation of JNK in EBSS, which induced autophagy and apoptosis under nutrient-deficient conditions (Wu et al., 2015).

## Phytochemicals Modulate Autophagy-Apoptosis Through ROS Signaling

ROS, such as O_2_^•−^, H_2_O_2_, and ^•^OH, are generated as metabolic by-products by biological systems; such generation may trigger detrimental as well as useful health outcomes (Covarrubias et al., 2008; Sena and Chandel, 2012). An optimum level of ROS is required for different biological processes, such as cell signaling, activation of proteins, immune function and transcriptional factors, and the regulation of apoptosis and differentiation (Rajendran et al., 2014). However, overproduction of ROS may have damaging effects on various proteins, lipids, and nucleic acids (Wu et al., 2013). Thus, an imbalance in ROS levels may be the cause of several diseases, such as cancer. Cellular ROS levels are also critical for cancer progression (Aggarwal et al., 2019). ROS-mediated DNA damage may play a critical role in the initiation and progression of carcinogenesis. Reversible DNA damage may allow an internal repair system to normalize the adverse effects of ROS. However, irreversible damage may not permit the proper functioning of the repair system. As a result, the cells undergo apoptosis, which has a considerable effect in cancer therapy (Aggarwal et al., 2019).

**FIGURE 6:**
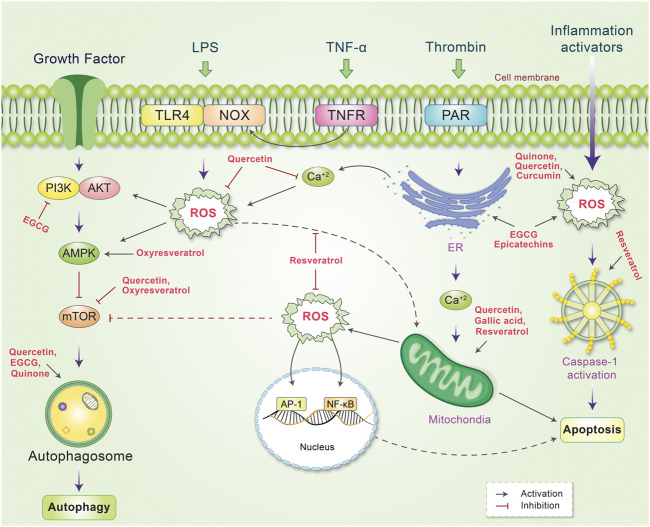
Schematic representation of the mechanism of action of phytochemicals and reactive oxygen species (ROS), which lead to the control of several signaling pathways. ROS is produced by several internal and external stimuli. Extranally, ROS is activated through growth factors, LPS, TNF-α, thrombin, and inflammation. Different phytochemicals have been found to scavenge or decrease cellular ROS level by inhibiting or stimulating their action. Internally, phytochemicals inhibit PI3K or mTOR, which activates autophagy and reduces ROS production. Some phytochemicals have also been found to activate mitochrondrial ROS production while other phytochemicals scavenge ROS and protect against DNA damage. ROS production mediated by ER and inflammation activators is also reduced by phytochemicals, which modulate the autophagy-apoptosis pathways.

As antioxidant phytochemicals can inhibit the growth of different cancer cells, they could serve as good candidates for anticancer therapy (Barrajón-Catalán et al., 2010; Sak, 2014). Depending on the concentration, exposure time, and ability of oxidative stress-inducing compounds, ROS signaling may act as an autophagic activator or apoptotic initiator in target cancer cells (Chirumbolo et al., 2018). EGCG is the most abundant polyphenol in green tea. EGCG has been found to induce apoptosis and autophagy in human mesothelioma cell death through prompting ROS (Satoh et al., 2013). Ha et al. represented that ROS generation is important in quercetin-meiated apoptotic cell death in Jurkat T cells has been targeted *via* BCL-XL antiapoptotic action protein (Ha et al., 2019). Epicatechins as shown to modulate autophagy and endoplasmic reticulum (ER) stress-induced apoptostic cell death of human various diseases (Zhang et al., 2020a). Gallic acid, 3,4,5-trihydroxy-benzoic acid found in red wine and grapes, acts as an auto-oxidation in addition to produce H_2_O_2_ and O_2_^−^ lead to intrinsic mitochondria-mediated apoptosis in prostate cancer cells (Russell Jr et al., 2012). Gallic acid prevents lung cancer cell growth *via* elevating ROS level as well as GSH depleting (Wang et al., 2016b). Gallic acid additionally encourages apoptosis through ROS-mediated activation of JNK pathways (Chen et al., 2013b). Oxidation of catechin-derived quinone has also been observed to result in anti-tumor activities in several human cancer cells through apoptotic as well as autophagic cell death *via* modulating ROS (Saibu et al., 2014; Lee et al., 2017). Thus, activation of oxidative stress signaling may not always be associated with unexpected side effects. A high dose of EGCG exerts pro-oxidant effects, which ultimately leads to autophagy activation and increased antitumor activity (Yang et al., 1998; Tsai et al., 2018; Bimonte et al., 2019). EGCG induces apoptosis in cancer cells through different mechanisms, including the suppression of PI3K/Akt signaling (Liu et al., 2016), reduction in mitochondrial membrane potential (Li et al., 2009) and expression of anti-apoptotic proteins, including Bcl-2, xIAP, and Bcl-xl (Wu et al., 2009). Previously, quercetin was found to promote ROS-stimulated apoptosis and autophagy in different cancers (Choi et al., 2008; Bi et al., 2016) by activating caspase-3 and inhibiting anti-apoptotic proteins, such as Bcl-2 and Bcl-xl. Additionally, quercetin reduces apoptosis in addition to decrease intervertebral disc degeneration through SIRT-mediated autophagy induction (Wang et al., 2020). In cancer cells, curcumin enhances TRAIL-induced apoptosis *via* ROS-mediated DR5 upregulation (Jung et al., 2005) and activates autophagy through the ROS-ERK1/2-p38 MAPK signaling pathway (Lee et al., 2011). Resveratrol has also been demonstrated to possess beneficial effects (Moni et al., 2018) it promotes apoptosis *via* ROS-dependent caspase activation (Shankar et al., 2007) and Bax/caspase-3 (Whitlock and Baek, 2012) and induces apoptosis associated with mitochondrial dysfunction in cancer cells (Lin et al., 2012). As depicted in Figure 6, phytochemicals are important modulators of cancer cell control owing to the autophagy-apoptosis pathways.

## Therapeutic Targets of Phytochemicals in Autophagy-Apoptosis Modulation for Cancer Prevention

Phytochemicals and naturally occurring compounds are well-known to ameliorate several human diseases owing to their pharmacological activities (Hannan et al., 2020; Rahman et al., 2020b). The most well-known anticancer agents, including taxol, resveratrol, vincristine, quercetin, vinblastine, tetrandrine, and arteannuin, modulate the autophagy-apoptosis pathway (Sun et al., 2019). Polyphenolic compounds and alkaloids are particularly dominant among all other cancer therapeutics (Newman and Cragg, 2016). Polyphenols play a greater role in apoptotic, autophagic, and cytostatic activities owing to their antioxidant properties, thereby serving as preventative cancer therapies (Focaccetti et al., 2019). Polyphenols can easily bind to cell membranes and trigger numerous signaling pathways, including caspases, epidermal growth factor (EGF), Bcl-2 family proteins, mitogen-activated protein kinase (MAPK), microRNAs (*miR*NAs), nuclear factor (NF)-κB, phosphatidylinositol-3-kinase PI3K/Akt/mTOR, and epidermal growth factor receptor (EGFR) (Sun et al., 2019). MicroRNAs (*miR*NAs) have also been demonstrated to regulate gene expression and are targeted as novel therapeutic approaches to control cancer; phytochemicals, such as resveratrol, silibinin, curcumin, genistein, and EGCG can be employed as apoptotic inducers, autophagy modulators, and cell cycle inhibitors (Lancon et al., 2012; Estrela et al., 2017; Jahanafrooz et al., 2018). *miR*NAs have been predicted to be critical for modulating cancer cell differentiation, invasion, proliferation, autophagy, and apoptosis *via* the regulation of oncogenic gene expression (Karius et al., 2012). Further, the MAPK and PI3K/Akt/mTOR signaling pathways have been shown to activate NF-κB in numerous cancer cell lines by modulating several phytochemicals in the autophagy-apoptosis pathway (Chao et al., 2017). Matrix metalloproteinase (MMP)-2 and MMP-9 modulate the autophagy-apoptosis pathway and control cancer through the action of different polyphenols (Balli et al., 2016).

Based on scientific evidence, phytochemicals present substantial anticancer potential for bench to bedside drug development. In fact, preclinical screening models can be used to assess their preliminary toxicity, safety, pharmacokinetics, and efficacy, which may serve as useful information for clinical trials (Zhang et al., 2020b). The preclinical efficacy of several phytochemicals, including ursolic acid, baicalein, genistein, 6-Shogaol, apigenin, thymoquinone, allicin, dicumarol, epigallocatechin, alpinumisoflavone, sulforaphane, curcumin, emodin, withaferin A, resveratrol, gingerol, physapubescin B, nimbolide, licochalcone A, glycyrrhizin, and hispidulin, has been demonstrated using numerous animal models (Choudhari et al., 2020). Despite several assessments of phytochemicals against cancer in the clinical trial setting, most trials continue to be in the early stage as numerous anti-cancer chemicals are currently being investigated. The most important phytochemicals under clinical trial investigation for various cancers include sulforaphane, resveratrol, lycopene, epigallocatechin, curcumin, and berberine; these phytochemicals aim to target the autophagy-apoptosis pathway (Choudhari et al., 2020).

## Limitations of Targeting the Autophagy-Apoptosis Crosstalk Using Phytochemicals in Anticancer Drug Development

Increasing evidence suggests that phytochemicals could exhibit anticancer effects by modulating various signaling pathways, such as autophagy and apoptosis (Figure 3). These two significant cellular pathways are largely responsible for determining the fate of cancerous cells (Su et al., 2013). However, such finding is mainly based on *in vitro* and preclinical *in vivo* investigations that may not necessarily guarantee clinical outcomes. Moreover, many phytochemicals target multiple signaling pathways that may be shared among multiple cellular systems. These multitargeted effects of phytochemicals may generate positive outcomes, but can also lead to unanticipated effects, thereby challenging the development of phytochemical-based anticancer drugs. Although many phytochemicals are not specific in their action and exert multitarget effects, it is uncertain whether their anticancer effects are autophagy-dependent or merely a response to mitigate the adverse conditions that support the survival of cells in the tumor microenvironment (Patra et al., 2020). Although autophagy and apoptosis are two critical cellular pathways in cancer biology, their specific roles remain unclear. However, because autophagy plays a critical role in cellular protein homeostasis and other quality control systems, modulating this crucial pathway may hamper cellular physiology. As autophagy is considered to be a double-edge sword, targeting this pathway may result in unprecedented outcomes.

## Conclusion and Future Perspectives

As the incidence of cancer increases on a daily basis, new strategies are being discovered to ensure this fatal disease is managed therapeutically. The major challenge in developing target-specific anticancer drugs is inextricably linked to the complexity of cancer pathobiology. Autophagy and apoptosis are two vital cellular pathways involved in cancer development and regulation. In addition, crosstalk is known to occur across signaling pathways, including those associated with autophagy and apoptosis. Many cancer types are becoming resistant to chemotherapy due to defects in signaling pathways, particularly apoptosis. As an alternative cell fate mechanism, autophagy could be explored for the development of target-specific anticancer drugs. Further investigations, both *in vitro* and *in vivo*, are however necessary to better understand cancer pathobiology, which will enable the full potential of autophagy-apoptosis-targeted drug design to be exploited.

Scientists have always been interested in the use of plant products and their derivatives as successful sources of anticancer therapeutics. In fact, there is increasing evidence suggesting the emerging anticancer potential of phytochemicals that modulate several signaling pathways, including autophagy and apoptosis. The anticancer effects of phytochemicals have been observed to be selective and specific to cancer cells, and involve the modulation of autophagy and apoptosis. As a result, many phytochemicals are promising sources of anticancer drugs. The most notable phytochemicals that have exerted their anticancer potential *in vitro* and *in vivo* through modulating the autophagy-apoptosis pathway (i.e., sulforaphane, resveratrol, lycopene, epigallocatechin, curcumin, and berberine) are currently being investigated in clinical trials for different cancer types.

Because autophagy plays a context-dependent role in cancer patients, targeting this crucial cellular pathway may not always be beneficial. Furthermore, several phytochemicals target multiple signaling pathways that may be shared among multiple cellular systems, thereby posing a challenge to the development of phytochemical-based anticancer drugs. This issue could however be resolved through *in vitro* and *in vivo* studies on phytochemical-mediated autophagy-apoptosis modulation. In addition, an integrated system pharmacology and computational approach could be employed to better understand the anticancer effects of phytochemicals. As the clinical application of phytochemicals is limited by their poor bioavailability, improvements can be achieved by employing nanotechnology-based drug delivery. Based on the highlights in this review, the potential as well as the challenges of phytochemical-mediated targeting of autophagy and apoptosis could unravel new approaches and strategies for the development of novel anticancer therapeutics to treat several cancer types.

Eventually, upcoming challenges as well as possible perspectives have been demonstrated in the hope of improving anticancer effectiveness in addition to accelerate the translational improvement of precise nanomedicine or nanotechnology for targeted cancer therapy based on autophagy-apoptosis pathway. Nanoparticle-based drug delivery systems (NDDSs) have been comprehensively used in the diagnosis, therapy, as well as cancer imaging because of their features of extraordinary cancer-targeting efficiency and low toxic properties. Nevertheless, because of the problems of poor patient prognosis, high variability, as well as multidrug resistance (MDR), NDDSs have currently been challenged remarkable experiments. Indeed, combined targets of nanoscience along with naturally occurring bioactive compounds are very attractive as well as developing rapidly in recent times in combination with conventional drugs for improving clinical outcomes. Therefore, it would be urgently required to necessary with designing novel treatment approaches to investigate in-depth the early diagnosis and pathogenesis of cancer thereby targeting phytochemicals through autophagy-apoptosis pathway.
